# Clinical utility and genetic landscape of exome sequencing in a large pediatric epilepsy cohort: Insights from a Turkish tertiary care center

**DOI:** 10.1002/epd2.70277

**Published:** 2026-07-03

**Authors:** Derya Karaer, Beste Kipçak Yüzbaşı, İpek Şahin, Olcay Güngör, Kadri Karaer

**Affiliations:** ^1^ Pamukkale University Faculty of Medicine Department of Medical Genetics Denizli Turkiye; ^2^ Pamukkale University, Faculty of Medicine Department of Pediatric Neurology Denizli Turkiye

**Keywords:** clinical exome sequencing, epilepsy, genetic diagnosis, genotype–phenotype correlation, next‐generation sequencing, pediatric epilepsy, whole‐exome sequencing

## Abstract

**Objective:**

To evaluate the diagnostic utility and genetic spectrum of next‐generation sequencing (NGS) in a large, well‐phenotyped cohort of Turkish pediatric patients with epilepsy of unknown etiology.

**Methods:**

Between January 2021 and December 2024, 250 children (115 female, 135 male) with unexplained epilepsy underwent either whole‐exome sequencing (WES; *n* = 104) or clinical exome sequencing (CES; *n* = 146). Variants were interpreted according to the American College of Medical Genetics and Genomics (ACMG) guidelines. Clinical data, including seizure semiology, neuroimaging, EEG findings, and treatment response, were retrospectively reviewed.

**Results:**

A definitive molecular diagnosis (pathogenic or likely pathogenic variants) was established in 89 patients (89/250, 35.6%). Including variants of uncertain significance (VUS) with strong phenotypic correlation raised the diagnostic yield to 57.6% (144/250). The genetic landscape was highly heterogeneous, involving 59 distinct genes. *SCN1A* was the most frequently implicated gene (*n* = 11, 12.4% of diagnosed cases), followed by *MECP2* and *PRRT2* (*n* = 5 each). Notably, the recurrent *PRRT2* c.649dup (p.Arg217Profs*8) frameshift variant was identified in four unrelated Turkish families. Inheritance patterns included autosomal dominant (56/89, 63%), autosomal recessive (20/89, 22%), and X‐linked (13/89, 15%). Diagnostic yields were comparable between WES (38/104, 36.5%) and CES (51/146, 35%) (*p* = 0.97). Neonatal‐onset epilepsy (*p* = 0.03) and the presence of autistic features (*p* = 0.04) were significantly associated with a positive genetic diagnosis.

**Conclusion:**

Our study confirms NGS as a first‐tier diagnostic tool in pediatric epilepsy, revealing a distinctive genetic architecture enriched with novel and population‐specific variants. These findings emphasize (1) a high diagnostic yield (∼36%) supporting first‐tier use of NGS; (2) a distinctive genetic architecture characterized by recurrent *PRRT2* variants and an elevated burden of autosomal recessive disorders, likely reflecting regional consanguinity patterns; and (3) a phenotype‐driven framework for prioritizing VUS re‐evaluation in clinical neurology practice. Such data from underrepresented populations are crucial for global genomic databases and directly inform precision medicine and genetic counseling.

**Key points**

- NGS provides 35.6% diagnostic yield in unexplained pediatric epilepsy.
- Recurrent *PRRT2* c.649dup may reflect either a known mutational hotspot or population‐specific enrichment.
- 22% of diagnoses were autosomal recessive, linked to consanguinity.
- Neonatal onset and autistic features predict higher diagnostic yield.
- Phenotype‐driven VUS reclassification improves clinical utility.

## 1 INTRODUCTION

Epilepsy affects 0.5%–1% of children worldwide and represents a clinically and genetically heterogeneous group of neurological disorders characterized by recurrent, unprovoked seizures.^1^, ^2^ Despite advances in neuroimaging and electroencephalography (EEG), a substantial proportion of pediatric epilepsy cases remain etiologically undiagnosed after standard clinical workup. The International League Against Epilepsy (ILAE) now emphasizes the integration of etiology into epilepsy classification, as identifying an underlying cause directly informs treatment selection, prognosis, and genetic counseling.^3^, ^4^

Over the past decade, next‐generation sequencing (NGS), particularly whole‐exome sequencing (WES) and targeted clinical exome sequencing (CES), has transformed the diagnostic pathway for neurogenetic disorders.^5^, ^6^ In pediatric epilepsy, NGS has yielded molecular diagnoses in 20%–50% of previously unexplained cases, with the highest yields observed in early‐onset, drug‐resistant, or syndromic presentations.^7^, ^8^ These diagnoses not only end the diagnostic odyssey for families but also enable precision therapeutics, such as avoiding sodium channel blockers in *SCN1A*‐related Dravet syndrome or initiating ketogenic diet in *SLC2A1*‐associated glucose transporter deficiency.^9^

However, most large‐scale genomic epilepsy studies have been conducted in populations of European or East Asian ancestry, potentially limiting the generalizability of findings to other ethnic groups.^10^ Emerging evidence indicates that the genetic architecture of epilepsy may differ across ancestries due to population‐specific allele frequencies, consanguinity patterns, and founder mutations.^11^ In Turkiye, where consanguineous marriages are relatively common, autosomal recessive disorders are more prevalent, and recurrent pathogenic variants may reflect local founder effects.^12^

In our current study, WES and CES were performed on a large cohort of 250 pediatric patients with varying ages of onset, diagnosed with epilepsy, and of unknown etiology (metabolic, immune and infectious related etiologies were ruled out). The primary aim was to determine the diagnostic efficiency of NGS in this Turkish pediatric cohort and to characterize the genetic spectrum, including recurrent variants and causative genes, in various epilepsy subgroups. In addition, a variant classification approach based on the American College of Medical Genetics (ACMG) guidelines has enabled the identification of causal variants such as pathogenic/possibly pathogenic (P/LP) and Variant of Uncertain Significance (VUS) in epilepsy‐related genes.

## 2 MATERIALS AND METHODS

### 2.1 Study design

This retrospective cohort study included 250 pediatric patients (aged ≤18 years) with epilepsy of unknown etiology (Patients with structural, infectious, immune‐mediated, or metabolic causes identified through neuroimaging, laboratory testing, or clinical history were excluded) referred from the Department of Pediatric Neurology to the Medical Genetics Outpatient Clinic at Pamukkale University Faculty of Medicine between January 2021 and December 2024. These patients were reclassified according to the ILAE updated 2025 classification by retrospectively re‐evaluating the records.

Inclusion criteria were: (1) The patient had a diagnosis of epilepsy. The diagnosis of epilepsy was supported by a detailed seizure history, ictal/interictal EEG recordings, and brain MRI. (2) Seizure onset before 18 years of age. (3) Normal basal metabolic screening (Tandem MS, amino acids, organic acids).

Exclusion criteria included: (1) Patients initially referred with a diagnosis of epilepsy but subsequently determined to have nonepileptic events based on detailed clinical history and follow‐up, such as febrile seizures or breath‐holding spells; (2) Written informed consent was obtained from legal guardians for genetic testing and the use of anonymized clinical and genomic data for research purposes.

### 2.2 Clinical phenotyping

Comprehensive phenotypic data were extracted from electronic medical records by two experienced pediatric neurologists (blinded to genetic results). Collected variables included:

- Age at seizure onset (categorized as neonatal: 0–28 days; infantile: 1–12 months; toddler: 13–36 months; childhood–juvenile: >36 months)
- Sex
- Family history of epilepsy or neurodevelopmental disorders
- Presence of intellectual disability/developmental delay (ID/DD)
- Behavioral features: autism spectrum disorder (ASD) traits, attention‐deficit/hyperactivity disorder (ADHD) (using DSM‐5 criteria), stereotypies, hyperactivity
- Neurological examination findings: abnormal muscle tone, micro/macrocephaly
- Brain MRI findings (available in 193/250, 77.2%)
- EEG results (available in 197/250, 78.8%): EEG recordings were performed as part of the initial diagnostic evaluation, typically within 6 months of seizure onset. The most representative EEG tracing (demonstrating interictal epileptiform abnormalities or ictal patterns correlating with the predominant seizure type) was selected for analysis in cases with multiple recordings.
- Epilepsy syndrome classification (e.g., developmental and epileptic encephalopathy [DEE])
- Seizure semiology was categorized as focal (F), unknown onset (U), generalized (G), or unclassified according to the updated 2025 ILAE framework,^13^ based on the most consistent patterns observed during the first year of epilepsy via EEG, caregiver reports, and clinical notes; however, 58 patients (23.2%) were designated as “unclassified” due to insufficient data.
- Seizure semiology describes the ictal event (e.g., generalized tonic–clonic, focal impaired awareness), whereas epilepsy syndrome classification (e.g., Dravet syndrome, developmental and epileptic encephalopathy) integrates seizure types with age of onset, EEG findings, developmental trajectory, and genetic etiology per ILAE criteria.
- Response to anti‐seizure medications (ASM), with drug resistance defined as failure of ≥2 appropriately chosen and dosed ASMs.^14^

### 2.3 Genomic DNA extraction and sequencing

Genomic DNA was extracted from peripheral blood leukocytes using the QIAamp DNA Blood Mini Kit (Qiagen, Hilden, Germany). DNA concentration and purity were assessed via spectrophotometry (NanoDrop 2000, Thermo Fisher Scientific).

Next‐generation sequencing was performed using two platforms:

#### 2.3.1 Whole‐exome sequencing (WES) (*n* = 104)

Library preparation with the Agilent SureSelect Human All Exon V7 kit, followed by paired‐end sequencing on the Illumina NovaSeq 6000 platform (mean coverage depth: ~100×; ≥97% of target bases covered at ≥20×).

#### 2.3.2 Clinical exome sequencing (CES) (*n* = 146)

Targeted sequencing of a curated panel of >4500 clinically relevant genes using an accredited diagnostic pipeline.

Sequence reads were aligned to the human reference genome (GRCh37/hg19). Variants were called using GATK best practices.

### 2.4 Bioinformatic analysis and variant interpretation

Variant annotation and filtering were performed using Sophia DDM (v5.10.14) and VarSome (v4.2). Filtering criteria included: Minor allele frequency (MAF) <1% in gnomAD; Predicted deleterious impact (e.g., missense, frameshift, nonsense, splice‐site); Presence in genes with established or definitive disease validity (per ClinGen). All candidate variants were classified according to the American College of Medical Genetics and Genomics/Association for Molecular Pathology (ACMG/AMP) guidelines.^15^ Only pathogenic (P) and likely pathogenic (LP) variants in genes with strong genotype–phenotype correlation was considered diagnostic. Variants of uncertain significance (VUS) were recorded but not included in the primary diagnostic yield.

A multidisciplinary team, including clinical geneticists, molecular biologists, and pediatric neurologists, reviewed all P/LP/VUS findings in the context of individual phenotypes.

### 2.5 Statistical analysis

Categorical variables (e.g., sex, ID/DD, drug resistance, autistic features) were compared between diagnostic subgroups using Fisher's exact test. Odds ratios (OR) with 95% confidence intervals (CI) were calculated where applicable. A two‐sided *p*‐value <0.05 was considered statistically significant. Analyses were performed using SPSS v28 and R v4.3.

## 3 RESULTS

### 3.1 Cohort characteristics and diagnostic yield

**FIGURE 1:**
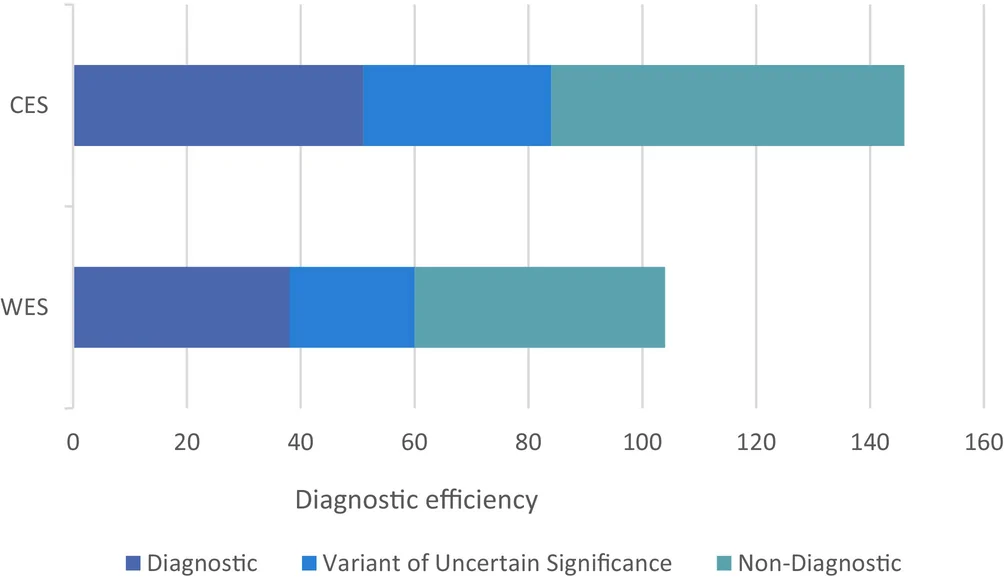
CES and WES methods diagnostic efficiency.

Of 250 patients (115 female, 135 male), a definitive molecular diagnosis (P or LP variant) was established in 89 (35.6%). An additional 55 patients (22%) carried VUS, and 106 (42.4%) remained undiagnosed. The diagnostic yield did not differ significantly between WES (38/104, 36.5%) and CES (51/146, 35%) (*p* = 0.94) (Figure 1).

Age at seizure onset showed marked variation in diagnostic yield: Neonatal: 8/15 (53.3%); infantile: 32/93 (34.4%); toddler: 11/43 (25.6%); childhood–juvenile: 38/99 (38.4%). Neurodevelopmental comorbidities were common: ID/DD: 165/250 (66.0%); autistic features: 10/250 (4.0%); ADHD: 12/250 (4.8%); abnormal muscle tone: 34/250 (13.6%). Microcephaly was observed in 34/250 (13.6%), macrocephaly in 7/250 (2.8%). A family history of epilepsy was present in 56/250 (22.4%). Treatment resistance was present in 28% (70/250) of the cohort, and developmental and epileptic encephalopathy (DEE) was present in 19.6% (49/250). In terms of seizure semiology, unclassified seizures constituted the largest clinical group, reflecting the inherent diagnostic challenges and phenotypic fluidity often encountered in early‐onset epilepsy cases. Generalized tonic–clonic seizures (GTC) were the second most frequent presentation, followed by focal impaired awareness seizures (Table 1).

**TABLE 1:** Clinical and demographic characteristics of the study cohort by diagnostic outcome.

	Total number of patients	Genetically diagnosed	VUS	Not diagnosed	*p*‐Value^a^
Total number of patients	250	89	55	106	
Gender (Female/Male)	115/135	45/44	23/32	47/59	
Seizure onset					
Neonatal	15	8	1	6	*p* = 0.03
Infantile	93	32	22	39	
Toddler	43	11	13	19	
Childhood–Juvenile	99	38	19	42	
Family history for epilepsy	56	14	18	24	
Intellectual disability/developmental delay (ID/DD)	165	60	35	70	*p* = 0.50
Head circumference					
Microcephaly	34	13	6	15	
Macrocephaly	7	4	‐	3	
Abnormal Brain MRI^b^	99/193	32	24	43	
Abnormal muscle tone	34	11	11	12	
Behavior	42	18	7	17	
Autistic behavior	10	7	1	2	*p* ≈ 0.04
ADHD	12	2	2	8	
Stereotypical movements	5	5	‐	‐	
Behavioral disorder	13	3	4	6	
Hyperactivity	2	1	‐	1	
Drug resistance	70	27	14	29	*p* = 0.46
Type of genetic testing					
Whole exome sequencing	104	38	22	44	
Clinical exome sequencing	146	51	33	62	
Developmental and epileptic encephalopathy (DEE)	49	25	11	13	
Classification of epileptic seizures^c^					
Focal (F)					
Focal preserved consciousness seizure (FPC)	6	3	‐	3	
Focal impaired consciousness seizure (FIC)	42	16	12	14	
Focal‐to‐bilateral tonic–clonic seizure (FBTC)	16	4	3	9	
Unknown whether focal or generalized (U)					
U‐Preserved consciousness seizure (PC)	1	1	‐	‐	
U‐ İmpaired consciousness seizure (IC)	7	3	2	2	
U‐Bilateral tonic–clonic seizure (BTC)	4	1	1	2	
Generalized (G)					
Absence seizures (AS)	14	2	6	6	
Generalized tonic–clonic seizure (GTC)	61	25	13	23	
Absence‐to‐tonic–clonic seizure (ATC)	1	1	‐	‐	
Other generalized seizures					
Generalized myoclonic seizure (GM)	1	‐	‐	1	
Generalized clonic seizure (GC)	5	2	‐	3	
Generalized negative myoclonic seizure (GNM)	‐	‐	‐	‐	
Generalized epileptic spasms (GES)	11	8	‐	3	
Generalized tonic seizure (GT)	13	5	4	4	
Generalized atonic seizure (GA)	2	‐	1	1	
Generalized myoclonic–atonic seizure (GMA)	8	‐	4	4	
Unclassified	58	18	9	31	

Abbreviation: ADHD: Attention‐deficit/hyperactivity disorder (DSM‐5 criteria).

^a^ *p*‐Value calculated using Fisher's exact test (two‐tailed); statistical significance defined as *p* < 0.05.

^b^ MRI data available for 193/250 patients (77.2%).

^c^ Seizure classification per ILAE 2025 operational criteria.^13^

### 3.2 Neuroimaging and neurophysiology

Brain MRI was abnormal in 99/193 (51.3%) of patients with available imaging. Among the genetically diagnosed patients with MRI data, 45.7% (37/81) had structural abnormalities (e.g., atrophy, white matter changes, cortical dysplasia).

EEG was available for 197 patients; epileptiform abnormalities were observed in 117 (59.4%). Of these, 53 (53/117, 45.3%) received a definitive genetic diagnosis, while 36 (45%) of the 80 patients with normal EEG received a genetic diagnosis, which was not statistically significant (*p* = 0.09).

### 3.3 Genetic findings and variant spectrum

**FIGURE 2:**
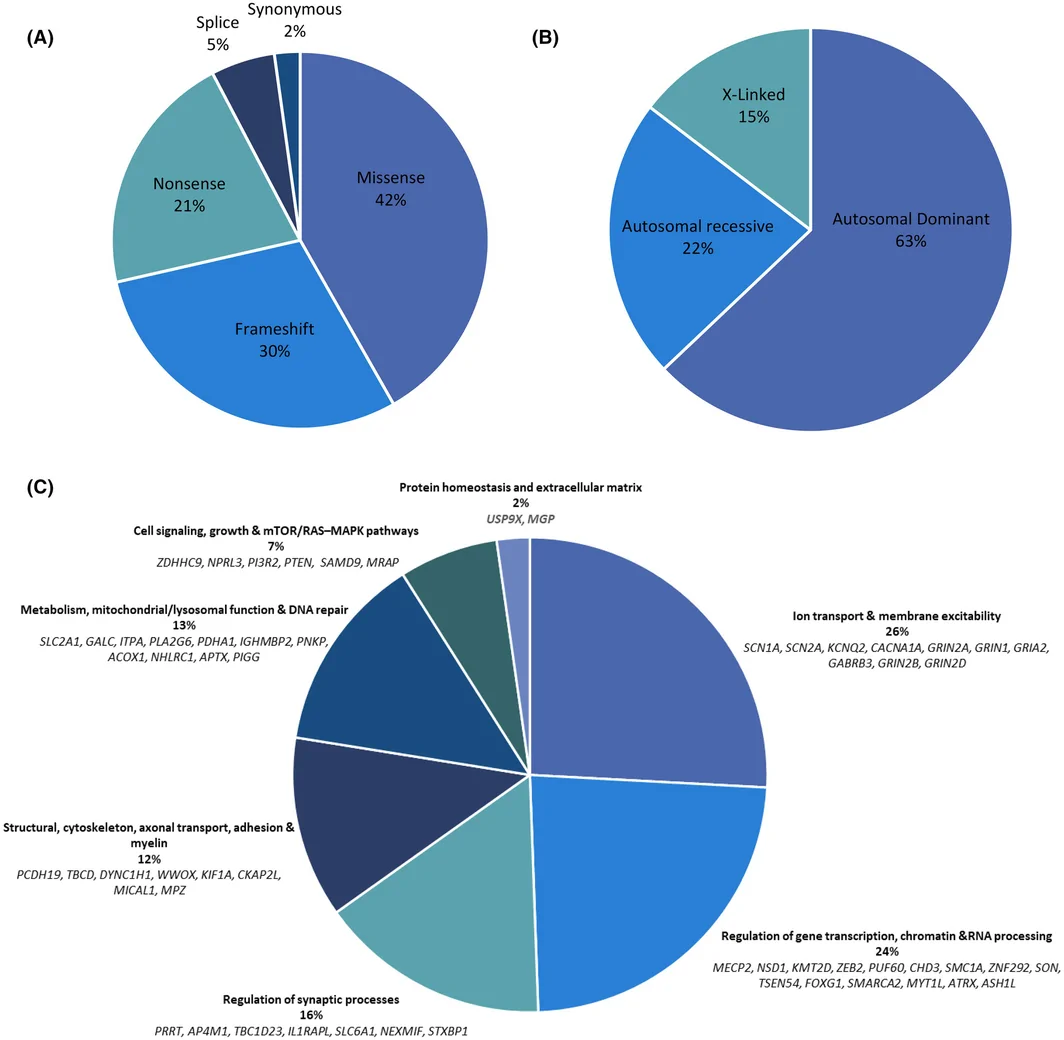
In cases with definitive molecular diagnosis. (A) Types of variants identified. (B) Observed inheritance patterns. (C) Distribution of identified genes according to pathway information.

In our cohort, a definitive molecular diagnosis was made in 89 (89/250, 35.6%) patients. The molecular profile of these patients included 83 different variants (LP/P) in 59 different genes, and the allelic variant types were as follows: Missense: 38 (42%); Frameshift: 27 (30%); Nonsense: 19 (21%); Splice site: 5 (5%); Synonym: 2 (2%) (Figure 2A). Thirty‐two of the detected variants were novel and had not been previously reported. The inheritance pattern of the patients was autosomal dominant (AD) in 63% (56/89), autosomal recessive (AR) in 22% (20/89), and X‐linked (XL) in 15% (13/89) (Figure 2B). The *SCN1A* gene, detected in 11 cases, represented the largest proportion among the genes with the most frequently identified variants. The majority of *SCN1A* variants were SNVs, often missense variants. Other frequently recurring genes included *PRRT2* (*n* = 5). Specifically, the *PRRT2* frameshift variant c.649dup (p.Arg217Profs*8) was found in four unrelated patients in this cohort, indicating a potentially recurring variant. Other genes, in order of frequency, were *KCNQ2* (*n* = 3), *SCN2A* (*n* = 2), *NSD1* (*n* = 2), *SLC2A1* (*n* = 2), and *ZEB2* (*n* = 2). In the AR inheritance model, *AP4M1* (*n* = 3), *TBC1D23* (*n* = 2), and *WWOX* (*n* = 2) were the most frequently encountered genes. Among the 9 genes showing X‐linked inheritance, the most common were *MECP2* (*n* = 5) and *PCDH19* (*n* = 2).

Furthermore, 53 promising VUS variants were identified in 51 different genes in 55 of our patients. Twenty‐six of these variants had not been previously reported. Although there was no evidence to elevate the classification of these rare VUS variants according to ACMG criteria, they were considered because they affect a conserved amino acid, may be deleterious, and may be associated with a range of diseases matching the patients' phenotype. Table 2 provides a comprehensive list of all identified genes and variants (LP/P/VUS). To elucidate the genetic pathogenesis of epilepsy, genes with variants (P/LP) identified in our cohort were divided into 7 different groups according to their function: cell signaling, growth, and mTOR/RAS–MAPK pathways; ion transport and membrane excitability; metabolism, mitochondrial/lysosomal function, and DNA repair; protein homeostasis and extracellular matrix; regulation of gene transcription, chromatin, and RNA processing; regulation of synaptic processes; and structural, cytoskeleton, axonal transport, adhesion, and myelin. The largest segment consisted of ion transport and membrane excitability genes (*SCN1A, SCN2A, KCNQ2, CACNA1A, GRIN2A, GRIN1, GRIA2, GABRB3, GRIN2B, GRIN2D*). The second largest segment was regulated by genes involved in transcription regulation, chromatin processing, and RNA processing (*MECP2, NSD1, KMT2D, ZEB2, PUF60, CHD3, SMC1A, ZNF292, SON, TSEN54, FOXG1, SMARCA2, MYT1L, ATRX, ASH1L*), while protein homeostasis and extracellular matrix genes (*USP9X, MGP*) were present in only two patients and had the lowest presence (Figure 2C). The gene group distribution in VUS was similar to LP/P variants, with the highest proportion found in ion transport and membrane excitability genes.

**TABLE 2:** Pathogenic, likely pathogenic, and select variants of uncertain significance identified in the cohort.

Case ID	Gender	Gene	Variant type	Nucleotide change	Predicted effect	Zyg	Novel/reported	ACMG criteria applied	ACMG Clas.	Seizure onset	Classification of epileptic seizures^a^	RTM
45	F	*SCN1A*	Missense	c.4222T>C	p.Trp1408Arg	Het	Novel	PM1, PP2, PP3, PM2	LP	Infantile	GTC	
64	M	*SCN1A*	Synonymous	c.1170G>A	p.Leu390=	Het	Reported ClinVar ID:946265	PS2, PM2, PP3	LP	Infantile	GT	R
85	F	*SCN1A*	Nonsense	c.1624C>T	p.Arg542*	Het	Reported ClinVar ID:189848	PVS1, PM2, PS2	P	Childhood	GTC	
104	F	*SCN1A*	Frameshift	c.1496del	p. Asn499Ilefs*45	Het	Novel	PVS1, PM2	LP	Childhood	FBTC	R
127	M	*SCN1A*	Nonsense	c.1702C>T	p.Arg568*	Het	Reported ClinVar ID:265254	PVS1, PM2, PS2	P	Infantile	Unclassified	
136	F	*SCN1A*	Missense	c.82C>T	p.Arg28Cys	Het	Reported ClinVar ID:206802	PS4, PM2, PM5, PP3PP2	P	Childhood	GTC	
165	F	*SCN1A*	Missense	c.3709T>C	p.Phe1237Leu	Het	Novel	PM1, PP2, PM5, PP3	LP	Childhood	FIC	
180	M	*SCN1A*	Missense	c.5132G>A	p.Gly1711Asp	Het	Novel	PM1, PP2, PM2, PM5, PP3	LP	Toddler	Unclassified	R
187	F	*SCN1A*	Frameshift	c.1655del	p.Pro552fs*6	Het	Novel	PVS1, PM2	LP	Infantile	GC	
206	M	*SCN1A*	Missense	c.4015G>T	p.Ala1339Ser	Het	Novel	PM1, PP2, PM2, PM5, PP3	LP	Childhood	PC	
238	M	*SCN1A*	Missense	c.2051C>T	p.Thr684Ile	Het	Reported ClinVar ID:643782	PM2, PP3, PP2	VUS	Infantile	GTC	
244	M	*SCN1A*	Frameshift	c.765_766del	p.Phe256Leufs*20	Het	Reported ClinVar ID:280823	PVS1, PS4, PM2	P	Infantile	Unclassified	R
47	F	*MECP2*	Missense	c.433C>T	p.Arg145Cys	Het	Reported ClinVar ID:11809	PS4, PS2, PM2, PM5, PM1, PP3	P	Toddler	Unclassified	R
101	F	*MECP2*	Missense	c.437C>G	p.Ser146Cys	Het	Reported ClinVar ID:143562	PS3, PS2, PM2, PM5, PM1, PP3	P	Childhood	FBTC	
128	F	*MECP2*	Nonsense	c.916C>T	p.Arg306*	Het	Reported ClinVar ID:11819	PVS1, PS2, PS3, PS4, PM2	P	Childhood	GTC	R
140	F	*MECP2*	Missense	c.509C>G	p.Thr170Arg	Het	Reported ClinVar ID:2135175	PS4, PM2, PM5, PM1, PP3	LP	Infantile	GTC	
245	F	*MECP2*	Nonsense	c.1200_1243del	p.Pro401*	Het	Reported ClinVar ID:143406	PVS1, PP1, PS2, PM2	P	Childhood	Unclassified	
58	F	*PRRT2*	Frameshift	c.649dup	p.Arg217Profs*8	Het	Reported ClinVar ID:65758	PVS1, PS3, PS4, PP1, PM2	P	Infantile	GTC	
99	F	*PRRT2*	Frameshift	c.649dup	p.Arg217Profs*8	Het	Reported ClinVar ID:65758	PSV1, PS4, PS3, PP1, PM2	P	Infantile	GTC	
100	F	*PRRT2*	Frameshift	c.649dup	p.Arg217Profs*8	Het	Reported ClinVar ID:65758	PVS1, PS4, PS3, PP1, PM2	P	Childhood	Unclassified	
197	M	*PRRT2*	Synonymous	c.1011C>A	p.Gly337=	Het	Reported *ClinVar ID:372477	PS2, PS4, BS1	P	Infantile	Unclassified	
247	M	*PRRT2*	Frameshift	c.649dup	p.Arg217Profs*8	Het	Reported ClinVar ID:65758	PVS1, PS3, PS4, PP1, PM2	P	Infantile	IC	
26	F	*PCDH19*	Missense	c.50C>T	p.Thr17Met	Het	Reported ClinVar ID:2434604	PM1, PP2, PM2	VUS	Toddler	GT	
89	M	*PCDH19*	Missense	c.1238G>T	p.Arg413Leu	Hemi	Novel	PM1, PP2, PM2, PP3	LP	Childhood	GT	R
130	F	*PCDH19*	Nonsense	c.1198G>T	p.Glu400*	Het	Novel	PVS1, PM2	LP	Infantile	GTC	R
65	M	*AP4M1*	Nonsense	c.1264C>T	p.Arg422*	Hom	Reported dbSNP ID: rs1440310254	PVS1, PM2	LP	Neonatal	FIC	
66	F	*AP4M1*	Nonsense	c.1264C>T	p.Arg422*	Hom	Reported dbSNP ID: rs1440310254	PVS1, PM2	LP	Childhood	Unclassified	
69	M	*AP4M1*	Nonsense	c.1012C>T	p.Arg338*	Hom	Reported ClinVar ID:209980	PVS1, PM3, PM2	P	Infantile	GTC	
71	M	*SCN2A*	Missense	c.4954G>A	p.Ala1652Thr	Het	Novel	PM1, PP2, PM2, PM5, PP3	LP	Childhood	Unclassified	
80	M	*SCN2A*	Missense	c.4418T>C	p.Ile1473Thr	Het	Reported ClinVar ID:427011	PS4, PM1, PP2 PM2, PM5, PP3	P	Childhood	GTC	
250	M	*SCN2A*	Missense	c.2300T>C	p.Ile767Thr	Het	Reported ClinVar ID:1318991	PM2, PP3, PP2	VUS	Infantile	Unclassified	
119	F	*KCNQ2*	Nonsense	c.2425C>T	p.Gln809*	Het	Reported ClinVar ID:813752	PVS1, PS4, PM2	P	Childhood	FBTC	R
152	M	*KCNQ2*	Missense	c.629G>C	p.Arg210Pro	Het	Reported ClinVar ID:280614	PS4, PM1, PP2, PM2, PM5, PP3	P	Neonatal	GTC	
219	M	*KCNQ2*	Missense	c.1037T>C	p.Phe346Ser	Het	Novel	PM1, PP2, PM2, PP3	LP	Neonatal	GC	
133	M	*CNTNAP2*	Missense	c.963C>G	p.Phe321Leu	Hom	Reported ClinVar ID:546436	PM2	VUS	Infantile	FIC	
134	M	*CNTNAP2*	Missense	c.963C>G	p.Phe321Leu	Hom	Reported ClinVar ID:546436	PM2	VUS	Infantile	GTC	
182	F	*CNTNAP2*	Missense	c.1560C>G	p.Cys520Trp	Hom	Novel	PM2, PP3	VUS	Childhood	Unclassified	
3	M	*TBC1D23*	Frameshift	c.743_744del	p.Gln248Argfs*43	Hom	Reported ClinVar ID:4293118	PVS1, PM3, PM2	P	Infantile	GTC	
4	M	*TBC1D23*	Frameshift	c.743_744del	p.Gln248Argfs*43	Hom	Reported ClinVar ID:4293118	PVS1, PM3, PM2	P	Toddler	FIC	
5	F	*TBCD*	Frameshift/ Missense	c.1575del/ c.2848C>T	p.His526Metfs*6/ p.Pro950Ser	Com.Het	Novel/ Novel	PVS1, PM2/PM2	LP/ VUS	Toddler	GTC	R
239	F	*TBCD*	Frameshift/ Missense	c.1575del/ c.2848C>T	p.H526Mfs*6/ p.P950S	Com.het	Novel/ Novel	PVS1, PM2/ PM2	LP/ VUS	Childhood	GT	R
9	M	*NSD1*	Frameshift	c.4858_4859del	p.Val1620ProfsTer27	Het	Novel	PVS1, PM2	LP	Infantile	GTC	
137	M	*NSD1*	Splice donor	c.4378+3_4378+6del	Splicing	Het	Reported ClinVar ID:565696	PS2, PP3, PM2	LP	Childhood	Unclassified	
10	M	*CACNA1A*	Missense	c.6743C>T	p.Ser2254Leu	Het	Reported ClinVar ID:1514365	PM2, PP3, PP2	VUS	Toddler	GTC	
20	F	*CACNA1A*	Frameshift	c.2045dup	p.Val683Argfs*98	Het	Reported ClinVar ID:2021246	PVS1, PM2, PP5	P	Neonatal	FPC	
13	M	*DYNC1H1*	Missense	c.3345G>C	p.Lys1115Asn	Hom	Novel	PM2, PP2	VUS	Neonatal	GT	R
98	F	*DYNC1H1*	Frameshift	c.947_948insGACAC	p.Phe316Leufs*9	Het	Novel	PVS1, PM2	LP	Infantile	GTC	R
15	F	*GRIN2A*	Missense	c.2327A>G	p.Asp776Gly	Het	Novel	PM2, PM5, PP3, PM1	LP	Childhood	BTC	R
179	F	*GRIN2A*	Missense	c.1985T>A	p.Val662Glu	Het	Novel	PM2, PM1, PP3	VUS	Toddler	AS	R
29	F	*WWOX*	Nonsense	c.790C>T	p.Arg264*	Hom	Reported ClinVar ID:241105	PVS1, PM3, PM2	P	Infantile	GES	R
51	M	*WWOX*	Nonsense	c.790C>T	p.Arg264*	Hom	Reported ClinVar ID:241105	PVS1, PM3, PM2	P	Neonatal	GES	
78	M	*GRIN1*	Missense	c.1913G>C	p.Gly659Ala	Het	Reported ClinVar ID:975942	PS2, PM1, PP2, PM2	LP	Infantile	GES	R
167	F	*GRIN1*	Missense	c.425C>T	p.Pro142Leu	Het	Novel	PM2, PP3, PP2	VUS	Infantile	Unclassified	
114	F	*SLC2A1*	Missense	c.697G>C	p.Gly233Arg	Het	Reported ClinVar ID:471348	PM1, PP2, PM2, PP3	LP	Infantile	Unclassified	
186	M	*SLC2A1*	Missense	c.695G>A	p.Arg232His	Het	Reported ClinVar ID:623683	PS4, PM1, PP2, PM2, PM5, PP3	P	Childhood	Unclassified	
131	F	*KMT2D*	Frameshift	c.2029del	p.Glu677Serfs*253	Het	Novel	PVS1, PM2	LP	Childhood	Unclassified	
144	F	*KMT2D*	Missense	c.8642G>A	p.Arg2881Gln	Het	Reported ClinVar ID:2891063	PM2, PP2, BP6	VUS	Toddler	GTC	
183	F	*GAD1*	Splice‐altering	c.1120‐493T>A	splicing	Hom	Novel	PM2	VUS	Infantile	GT	
184	F	*GAD1*	Splice‐altering	c.1120‐493T>A	splicing	Hom	Novel	PM2	VUS	Infantile	GT	
202	F	*RORB*	Missense	c.334A>T	p.Ser112Cys	Het	Reported ClinVar ID:1997337	PM2	VUS	Childhood	GMA	R
222	F	*RORB*	Missense	c.835C>T	p.Arg279Trp	Het	Reported ClinVar ID:1013334	PM2, PP3	VUS	Toddler	AS	
18	M	*ZEB2*	Missense	c.3133C>A	p.His1045Asn	Het	Novel	PM1, PP2, PM2 PM5, PP3	LP	Childhood	FIC	
223	F	*ZEB2*	Missense	c.898C>T	p.His300Tyr	Het	Reported ClinVar ID:3984323	PM2, PM5, PP3 PP2	LP	Childhood	FIC	
2	F	*GALC*	Missense	c.857G>A	p.Gly286Asp	Hom	Reported ClinVar ID:30619	PM3, PS3, PM1, PM2, PM5, PP3	P	Neonatal	GTC	R
6	M	*WDR81*	Missense	c.2206C>T	p.Leu1939Phe	Hom	Novel	PM2	VUS	Infantile	GTC	R
7	M	*SACS*	Missense	c.829A>G	p.Met424Val	Hom	Reported dbSNP ID: rs1286940495	PM2	VUS	Infantile	GTC	
12	M	*GLB1*	Missense	c.110G>A	p.Ser37Asn	Hom	Novel	PM1, PM2	VUS	Infantile	FIC	
14	F	*PTPN11*	Missense	c.164A>G	p.Lys55Arg	Het	Reported ClinVar ID:1397955	PM1, PP2, PM2	VUS	Childhood	Unclassified	
16	F	*GRIA2*	Missense	c.1939G>C	p.Val647Leu	Het	Reported ClinVar ID:929842	PS2, PM1, PP2, PM2, PM5	P	Neonatal	FIC	R
19	M	*IL1RAPL*	Nonsense	c.148C>T	p.Arg50*	Hemi	Novel	PVS1, PM3, PM2	P	Childhood	FPC	
21	F	*RELN*	Missense	c.6976G>A	p.Asp2326Asn	Het	Reported ClinVar ID:651420	PM2, BP6	VUS	Childhood	IC	
23	F	*PUF60*	Frameshift	c.1263del	p.Glu422Argfs*13	Het	Novel	PVS1, PM2	LP	Childhood	FBTC	
24	F	*CACNA1B*	In‐frame Deletion	c.5823_5831del	p.Arg1943_Gln1945del	Het	Novel	PM2, PM4	VUS	Infantile	FIC	R
27	F	*ITPA*	Splice donor	c.‐214+1G>A	splicing	Het	Reported ClinVar ID:646228	PVS1, PM3, PM2	P	Toddler	GTC	
28	M	*MTHFS*	Missense	c.157T>C	p.Ser53Pro	Hom	Novel	PM2	VUS	Childhood	FBTC	R
31	M	*SCN5A*	Missense	c.4009G>A	p.Val1337Ile	Het	Reported ClinVar ID:918378	PM2, PM1, PP3	VUS	Infantile	FIC	
32	M	*CHD3*	Nonsense	c.5099C>G	p.Ser1641*	Het	Novel	PVS1, PM2	LP	Infantile	FIC	
34	F	*GALNT2*	Missense	c.1084C>T	p.Arg362Trp	Hom	Reported dbSNP ID: rs774689179	PM2, PP3	VUS	Childhood	IC	
35	M	*KCNQ1*	İntronic	c.1033‐85A>G	‐	Het	Novel	PM2	VUS	Toddler	Unclassified	
36	M	*ZDHHC9*	Nonsense	c.892C>T	p.Arg298*	Hemi	Reported ClinVar ID:2228665	PVS1, PM3, PM2	P	Childhood	IC	
37	F	*PLA2G6*	Missense	c.2251G>A	p.Glu751Lys	Hom	Reported ClinVar ID:2138447	PM3, PM1, PM2	LP	Toddler	GT	
38	M	*KIF1A*	Missense	c.296C>T	p.Thr99Met	Het	Reported ClinVar ID:30169	PS2, PP2, PM2 PM5, PP3	P	Childhood	Unclassified	
39	F	*USP9X*	Splice site	Intron 24 c.3684+2T>G	splicing	Het	Novel	PVS1, PM2	LP	Infantile	GTC	R
40	M	*GABRB3*	Missense	c.867C>G	p.Ile289Met	Het	Novel	PP2, PM2, PP3	LP	Infantile	FIC	R
44	M	*CHRNA4*	Missense	c.1453C>T	p.Arg485Trp	Het	Reported ClinVar ID:1003101	PM2	VUS	Infantile	GMA	R
48	F	*SMC1A*	Missense	c.1193G>A	p.Arg398Gln	Het	Reported ClinVar ID:159938	PS4, PM2, PM5, PP3, PP2	LP	Toddler	GTC	
49	M	*KATNB1*	Missense	c.1835G>A	p.Arg612Lys	Hom	Novel	PM2, PP3	VUS	Childhood	FIC	
52	F	*ZNF292*	Frameshift	c.6305_6306del	p.Leu2102Argfs*53	Het	Novel	PVS1, PM2	LP	Infantile	GES	
57	M	*FGFR2*	Missense	c.532C>T	p.Arg178Cys	Het	Reported ClinVar ID:2226118	PM2, PP3, PP2	VUS	Infantile	FIC	
61	M	*RHOBTB2*	Deletion	c.2059_2061del	p.Lys709del	Het	Novel	PM2, PM4	VUS	Childhood	GA	R
67	F	*PDHA1*	Missense	c.788G>C	p.Arg301Pro	Het	Reported ClinVar ID:1184474	PM2, PM5, PP3, PP2	LP	Infantile	GTC	R
68	F	*IGHMBP2*	Missense	c.1540G>A	p.Glu514Lys	Hom	Reported ClinVar ID:9112	PM3, PM2, PP3	P	Infantile	FIC	
75	F	*CACNA2D2*	Missense	c.1046A>T	p.Asn349Ile	Hom	Novel	PM2, PP3	VUS	Infantile	FIC	
76	M	*KIF5C*	Missense	c.929A>G	p.Asn310Ser	Het	Novel	PM2, PP3, PP2	VUS	Childhood	GTC	
77	F	*CKAP2L*	Frameshift	c.981_982del	p.Arg327Serfs*16	Hom	Novel	PVS1, PM2	LP	Childhood	Unclassified	
79	F	*PNKP*	Frameshift	c.1253_1269dup	p.Thr424Glyfs*49	Hom	Reported ClinVar ID: 4847	PVS1, PM3, PP1, PS3, PM2	P	Childhood	GT	
81	F	*NPRL3*	Frameshift	c.511del	p.Leu171Trpfs*46	Het	Novel	PVS1, PM2	LP	Infantile	GES	R
83	M	*MGP*	Nonsense	c.43del	p.Val15*	Hom	Reported ClinVar ID:14341	PVS1, PM2, PP5	P	Infantile	FIC	
84	M	*PIK3R2*	Missense	c.1117G>C	p.Gly373Arg	Het	Reported *ClinVar ID:39808	PS1, PM2	LP	Infantile	GES	
86	M	*DDX3X*	Missense	c.1272A>C	p.Glu424Asp	Hemi	Novel	PM1, PM2	VUS	Childhood	GTC	
87	M	*GRIN2B*	Missense	c.2453T>C	p.Met818Thr	Het	Reported ClinVar ID:245960	PS2, PS3, PP2, PM2, PM5, PP3	P	Infantile	GES	
88	F	*PTEN*	Missense	c.611C>G	p.Pro204Arg	Het	Novel	PM1, PP2, PM2, PM5, PP3	LP	Childhood	IC	
92	M	*SON*	Frameshift	c.5753_5756del	p.Val1918Glufs*87	Het	Reported ClinVar ID:252929	PVS1, PS2, PM2	P	Infantile	FIC	
94	M	*YEATS2*	Missense	c.2738C>T	p.Ala913Val	Het	Reported dbSNP ID: rs769948328	PM2, PP3	VUS	Childhood	AS	
95	M	*RNASEH2A*	Missense	c.872G>A	p.Arg291His	Hom	Reported ClinVar ID:126401	PM2	VUS	Childhood	FIC	
96	M	*TRIO*	Missense	c.8897G>A	p.Gly2966Glu	Het	Reported dbSNP ID: rs903212005	PM2, PP2	VUS	Toddler	FBTC	
97	M	*HCFC1*	Missense	c.1024G>A	p.Gly342Ser	Hem	Novel	PM2, PP2	VUS	Childhood	Unclassified	
102	F	*ACOX1*	Nonsense	c.768T>A	p.Tyr256*	Het	Novel	PVS1, PM2	LP	Neonatal	FIC	
103	M	*TSEN54*	Missense	c.919G>T	p.Ala307Ser	Hom	Reported ClinVar ID:2120	PM3, PP1, PM2	P	Toddler	GTC	R
106	F	*HGSNAT*	Missense	c.1277G>A	p.Gly426Glu	Hom	Novel	PM2, PP3	VUS	Childhood	FIC	R
107	M	*FOXG1*	Frameshift	c.460dup	p.Glu154Glyfs*301	Het	Reported ClinVar ID:95268	PVS1, PS4, PM2	P	Childhood	FIC	R
109	M	*MICAL1*	Nonsense	c.2910G>A	p.Trp970*	Het	Reported ClinVar ID:2030661	PVS1, PM2	LP	Childhood	AS	
110	M	*SMARCA2*	Missense	c.3449C>T	p.Pro1150Leu	Het	Novel	PP2, PM2, PP3	LP	Infantile	GTC	
112	M	*SLC6A1*	Missense	c.863C>T	p.Ala288Val	Het	Reported ClinVar ID:192372	PS3, PS2, PP2, PM2, PM5, PP3, PP5	P	Childhood	FIC	
113	F	*NHLRC1*	Missense	c.436G>A	p.Asp146Asn	Hom	Reported ClinVar ID:162618	PM3, PP1, PS3, PM2, PM5, PM1	P	Childhood	GTC	
115	M	*KCNQ5*	Missense	c.1810G>A	p.Gly623Ser	Het	Reported *ClinVar ID:1390052	PM2, PP3, PP2	VUS	Toddler	GTC	
121	M	*SCN11A*	Nonsense	c.3448C>T	p.Arg1150*	Het	Reported ClinVar ID:967210	PM2	VUS	Toddler	AS	R
126	F	*DOCK7*	Missense	c.2195C>G	p.Thr732Arg	Hom	Reported ClinVar ID:1916429	PM2, PP2	VUS	Infantile	FIC	R
135	F	*NEXMIF*	Frameshift	c.1352_1359del	p.Asp451GlyfsTer2	Het	Novel	PVS1, PM2	LP	Toddler	ATC	R
138	M	*KDM5C*	Missense	c.56C>A	p.Pro19His	Hemi	Novel	PM2, PP3, PP2	VUS	Toddler	GTC	
141	M	*SCN9A*	Missense	c.2734G>A	p.Asp912Asn	Het	Reported ClinVar ID:1387587	PM2, PP3	VUS	Childhood	AS	
142	F	*MYT1L*	Frameshift	c.812dup	p.His272Thrfs*11	Het	Novel	PVS1, PM2	LP	Childhood	GTC	
145	M	*SYN1*	Missense	c.1966C>A	p.Pro656Thr	Hemi	Novel	PM2, BP4	VUS	Childhood	AS	
153	M	*SCN1B*	Missense	c.538C>T	p.Leu180Phe	Het	Reported ClinVar ID:1371605	PM2	VUS	Childhood	Unclassified	R
156	M	*ATRX*	Splice site	c.7201‐2A>G	Splicing	Hem	Reported ClinVar ID:11737	PVS1, PM2, PP5	LP	Childhood	Unclassified	
162	F	*AMPD2*	Missense	c.813G>A	p.Met271Ile	Hom	Novel	PM2, PP2	VUS	Toddler	Unclassified	
169	F	*SYNGAP1*	Missense	c.3677A>G	p.Gln1226Arg	Het	Novel	PM2, PP2	VUS	Infantile	GTC	
171	M	*APTX*	Frameshift	c.940_956del	p.Lys314Serfs*2	Hom	Reported ClinVar ID:870836	PVS1, PM2, PP5	LP	Childhood	Unclassified	
172	M	*SAMD9*	Nonsense	c.4299del	p.Leu1434*	Het	Reported dbSNP ID: rs1365970571	PVS1, PM2	LP	Toddler	FIC	
178	M	*CLPB*	Missense	c.422C>A	p.Ala141Asp	Hom	Novel	PM2	VUS	Infantile	GTC	
181	M	*KCNT1*	Missense	c.274G>A	p.Val92Ile	Het	Reported ClinVar ID:804967	PM2	VUS	Infantile	GMA	R
188	M	*PIGG*	Nonsense	c.1897C>T	p.Arg633*	Hom	Reported ClinVar ID:2871376	PVS1, PM2, PP5	P	Toddler	Unclassified	
191	F	*KAT8*	Missense	c.3G>C	p.Met1Ile	Het	Novel	PM2	VUS	Toddler	GMA	R
195	F	*KCNJ10*	Missense	c.896C>A	p.Ser299Tyr	Hom	Novel	PM2, PP3	VUS	Infantile	GTC	
196	F	*CLCN4*	Missense	c.1673C>G	p.Thr558Ser	Het	Novel	PM2, PM1, PP2, PP3	VUS	Toddler	FBTC	
199	M	*ASH1L*	Frameshift	c.914_915del	p.V305Efs*13	Het	Novel	PVS1, PM2	LP	Childhood	AS	
200	M	*MRAP*	splice donor	c.106+1delG	splicing	Hom	Reported ClinVar ID:444068	PVS1, PM3, PM2	P	Childhood	FIC	
207	M	*CPA6*	Missense	c.799G>A/ c.619C>G	p.Gly267Arg/ p.Gln207Glu	Com.Het	Reported	PM2, BS2, PP3, BP6/PM2, BS2, BP6	VUS/VUS	Infantile	BTC	
217	F	*GRIN2D*	Frameshift	c.120del	p.Gly42Alafs*41	Het	Novel	PVS1, PM2	LP	Infantile	GES	R
224	F	*SETD1A*	Missense	c.1010C>T	p.Ser337Phe	Het	Reported dbSNP ID: rs776546240	PM2	VUS	Childhood	FIC	
225	M	*STXBP1*	Missense	c.1652G>A	p.Arg551His	Het	Reported ClinVar ID:566474	PS2, PP2, PM2, PM5, PP3	P	Infantile	GTC	R
230	M	*MPZ*	Missense	c.106A>T	p.Arg36Trp	Het	Reported ClinVar ID:221065	PS4, PP1, PS3, PM1, PM2, PM5	P	Childhood	FPC	R
231	F	*MACF1*	Missense	c.21454C>T	p.Arg7152Trp	Het	Reported dbSNP ID: rs768877074	PM2, PP3, PP2	VUS	Infantile	FIC	
248	M	*CLCN2*	Missense	c.703C>T	p.Arg235Trp	Het	Reported dbSNP ID: rs199782817	PM2, PP3, PM5	VUS	Childhood	Unclassified	

*Note*: Full table includes all 98 P/LP and 47 clinically relevant VUS cases; only representative entries shown.

Abbreviations: AD, autosomal dominant; AR, autosomal recessive; ATC, absence‐to‐tonic–clonic; BTC, bilateral tonic–clonic; Com.Het, compound heterozygous; FBTC, focal‐to‐bilateral tonic–clonic; FIC, focal impaired consciousness; GC, generalized clonic; GES, generalized epileptic spasms; GMA, generalized myoclonic–atonic; GT, generalized tonic; GTC, generalized tonic–clonic; Hemi, hemizygous; Het, heterozygous; Hom, homozygous; IC, impaired consciousness; LP, likely pathogenic; P, pathogenic; PC, preserved consciousness; RTM, Response to treatment; VUS, variant of uncertain significance; XL, X‐linked; Zyg, zygosity.

^a^ Seizure classification follows ILAE 2025 operational criteria.^13^ Epilepsy syndrome diagnoses (e.g., Dravet syndrome, DEE) were assigned based on comprehensive clinical evaluation including seizure types, EEG, neuroimaging, and developmental history. FLBFR = Drug‐resistant: Failure of ≥2 appropriately chosen and dosed anti‐seizure medications.^14^ Cases marked with ‘R’ include the 12 patients in whom genetic diagnosis directly informed therapeutic decisions (see Section 3.5).

### 3.4 Clinical determinants of diagnosis

Statistical analysis revealed several clinical features associated with a higher probability of reaching a definitive molecular diagnosis (detection of P/LP variants):

#### 3.4.1 Neonatal seizure onset

Patients with neonatal epilepsy onset showed significantly higher diagnostic yield (8/15, 53.3%) compared to other age groups (range 11/43, 25.6% vs. 38/99, 38.4%), confirming that early onset is a strong predictor of genetic etiology (*p* = 0.03).

#### 3.4.2 Autistic features

The presence of autistic behaviors was significantly associated with a positive genetic diagnosis (P/LP) (7/10, 70.0% vs. 82/240, 34.1%; OR = 5.6, 95% CI: 1.2–25.7; *p* ≈ 0.04).

#### 3.4.3 Neurodevelopmental delay (ID/DD)

Although a high proportion of patients with a definitive diagnosis (60/165, 36.4%) had ID/DD, this proportion was not statistically significant when compared to patients without this characteristic (29/85, 34.1%) (*p* = 0.50).

#### 3.4.4 Drug resistance

No significant difference in diagnostic yield was observed between drug‐resistant (27/70, 38.6%) and non‐drug‐resistant (62/180, 34.4%) patients (*p* = 0.46).

#### 3.4.5 Neuroimaging and EEG

Although abnormal MRI findings were common among definitively diagnosed cases (32 out of 73 patients with available MRI data had abnormal MRI findings, 43.8%), structural abnormalities were not a statistically significant predictor of genetic diagnosis in this cohort (*p* > 0.05), possibly due to the inclusion of patients with acquired structural lesions. In the cohort (*n* = 197) that had access to EEG imaging, 59.4% (117/197) had abnormal EEG findings, and 45.3% (53/117) of these were diagnosed with a definitive molecular diagnosis.

### 3.5 Clinical utility of genetic diagnosis

In 12 of 89 diagnosed patients (13.5%), genetic results directly informed therapeutic decisions: (1) *SCN1A*‐positive cases (*n* = 9) avoided sodium channel blockers (e.g., carbamazepine, lamotrigine) to reduce seizure exacerbation risk; (2) *SLC2A1* variants (*n* = 2) prompted ketogenic diet trials with subsequent seizure reduction; (3) *MECP2* diagnosis (*n* = 1) enabled Rett syndrome‐specific surveillance including scoliosis monitoring and cardiac screening. Of these 12 patients, 6 were drug‐resistant (indicated with ‘R’ in Table 2), highlighting how genetic diagnosis can guide precision therapy in refractory cases.

## 4 DISCUSSION

This study reports a diagnostic yield of 35.6% (89/250) using next‐generation sequencing (NGS) in a large, unselected Turkish pediatric epilepsy cohort of unknown etiology a rate that aligns with or exceeds recent international benchmarks.^7^, ^16^, ^17^, ^18^, ^19^, ^20^ When clinically correlated variants of uncertain significance (VUS) are included, the potential diagnostic yield rises to 57.6% (144/250), directly informing precision therapeutics in 13.5% of diagnosed cases (e.g., sodium channel blocker avoidance in *SCN1A*, ketogenic diet initiation in *SLC2A1*). Beyond confirming NGS as a first‐tier diagnostic modality, our findings offer critical insights into genetic architecture, population‐specific variants, and clinical‐actionable implications in early‐onset epilepsy.

### 4.1 Genetic landscape and population‐specific architecture

The genetic spectrum in our cohort was remarkably heterogeneous, with 59 different genes and 83 variants (136 different variants when VUSs are included) playing a role in definitive diagnoses. This mirrors the known complexity of pediatric epilepsy but also reveals distinct population‐level patterns. Notably, the recurrent identification of the *PRRT2* c.649dup variant in four unrelated families may reflect either a known mutational hotspot or a potential population‐specific enrichment in the Turkish cohort. The hypothesis that would require haplotype analysis and comparison with population frequency databases for confirmation. Infantile onset seizures were observed in 3 of the 4 patients; this frameshift variant is a well‐established hotspot for benign familial infantile epilepsy (BFIE) and paroxysmal kinesigenic dyskinesia (PKD),^18^, ^21^ yet its recurrence in our cohort may reflect regional haplotype conservation, possibly driven by high consanguinity rates (prevalence ~20% in Turkiye). This observation underscores the need for population‐tailored variant databases and highlights how underrepresented cohorts can reveal geographically enriched pathogenic alleles.

Given the elevated burden of autosomal recessive disorders (22% of diagnoses) in our cohort likely reflecting regional consanguinity patterns we recommend that NGS panels deployed in populations with high consanguinity rates should prioritize comprehensive coverage of AR epilepsy genes, particularly those associated with developmental and epileptic encephalopathy (e.g., *AP4M1, TBC1D23, WWOX*).^2^, ^22^

While some pediatric epilepsy cohort studies report that women may be more likely to receive a diagnosis than men, almost twice as often^23^, ^24^; there are also large cohort studies reporting no difference between genders.^19^, ^25^ In our patient population, no statistically significant difference was found between female and male patients in terms of diagnosis rates (*p* > 0.05).

### 4.2 Genotype–phenotype correlations and DEE spectrum

Forty‐nine patients (49/250, 19.6% of the entire cohort; 25 with P/LP variants, 11 with VUS, and 13 undiagnosed) were clinically classified as having developmental and epileptic encephalopathy (DEE), confirming that DEE constitutes a substantial proportion of severe pediatric epilepsy cases. Notably, one male patient (Case 89) carried a hemizygous *PCDH19* missense variant (c.1238G>T, p.Arg413Leu). *PCDH19*‐related epilepsy typically follows an atypical X‐linked inheritance pattern, wherein heterozygous females are affected while hemizygous males are usually spared due to a “cellular interference” mechanism requiring coexistence of *PCDH19*‐expressing and nonexpressing neurons. Rare affected males have been reported and may result from: (1) somatic mosaicism confined to brain tissue; (2) skewed X‐chromosome inactivation in carrier mothers with transmission to sons; or (3) dominant‐negative effects of specific missense changes.^26^, ^27^ Parental samples were unavailable to confirm inheritance or mosaicism; however, this case aligns with prior reports of mosaic or hemizygous *PCDH19* males with epilepsy. It is important to distinguish between seizure semiology (the ictal event) and epilepsy syndrome diagnosis (the broader clinical entity). For example, *SCN1A* variants were associated with diverse seizure semiologies (focal‐to‐bilateral tonic–clonic (fbtc), generalized tonic–clonic (GTC), unclassified) but consistently met criteria for Dravet syndrome or DEE based on early onset, developmental regression, and EEG findings. Similarly, *KCNQ2* variants presented with neonatal GTC or Generalized Clonic (GC) seizures, consistent with *KCNQ2*‐related neonatal epilepsy. This distinction underscores that seizure semiology represents a single dimension of the phenotype, whereas syndrome diagnosis integrates multiple clinical domains over time. The predominance of ion channel genes (*SCN1A*, *SCN2A*, *KCNQ2*, *CACNA1A*) in this subgroup is consistent with global reports, but our data add granularity: all 11 *SCN1A*‐positive patients exhibited severe phenotypes, with 9 meeting clinical criteria for Dravet syndrome. Crucially, 3 patients initially classified as having “unclassified seizures” were later re‐evaluated and recognized as having Dravet‐like features only after genetic diagnosis highlighting how molecular data can refine phenotypic classification. *KCNQ2*‐related neonatal epilepsy (*n* = 3) presented with FBTC, GTC or GC seizures in the first week of life. Two patients with *WWOX* p.Arg264* (Cases 29, 51) exhibited generalized epileptic spasms, developmental regression, and abnormal MRI—classic for *WWOX*‐associated DEE28. A particularly noteworthy finding was the identification of a novel homozygous missense variant in *DYNC1H1* (Case 13, p.Lys1115Asn) in a neonate presenting with GT seizures and lissencephaly. While *DYNC1H1* is traditionally associated with autosomal dominant neurodevelopmental disorders, our identification of a homozygous variant suggests a recessive inheritance pattern, thereby expanding the known allelic and clinical spectrum of the gene beyond its typical dominant manifestations. Two siblings with *TBC1D23* frameshift variants (Cases 3–4) displayed corpus callosum agenesis, pontocerebellar hypoplasia, and focal impaired awareness seizures—consistent with Pontocerebellar Hypoplasia Type 11 (PCH11) but with earlier seizure onset than previously reported. Conversely, non‐DEE‐associated variants (e.g., *PRRT2*, *MECP2*, *KMT2D*) were often linked to milder or syndromic presentations, such as Rett‐like features, intellectual disability without regression, or self‐limited familial epilepsies. This phenotypic stratification supports the dual utility of NGS: not only for diagnosing monogenic DEEs but also for subtyping complex neurodevelopmental disorders where epilepsy is a secondary feature.

### 4.3 Clinical predictors of diagnostic yield

While intellectual disability (ID/DD) and drug resistance were highly prevalent (66% and 28%, respectively), neither significantly predicted a genetic diagnosis in our cohort (*p* > 0.05). This challenges the assumption that severe phenotypes uniformly increase diagnostic yield and suggests that phenotypic severity alone is a triage criterion for NGS.

The timing of seizure onset serves as a critical indicator for genetic etiology; neonatal and infantile onset are strongly associated with a higher likelihood of molecular diagnosis. A prospective national cohort study found that presentation before 6 months of age is significantly associated with a genetic diagnosis, with single‐gene disorders accounting for a quarter of seizure disorders in children presenting before 36 months. Meta‐analytical data further supports this, showing a pooled diagnostic yield of 29.3% specifically for the neonatal/infantile subgroup.^28^ Furthermore, clinical comorbidities such as intellectual disability (ID) and developmental delay (DD) significantly increase diagnostic success rates. While the general diagnostic yield in patients with autistic features is approximately 17.1%, this figure rises to 24.6% when autistic features co‐occur with ID or DD.^29^ These findings align with emerging evidence linking specific genes, such as *PCDH19*, to epilepsy–autism comorbidity, particularly in early childhood. Collectively, these data reinforce the concept that early‐onset epilepsies with neurodevelopmental features are frequently monogenic, warranting primary genetic investigation in these cohorts.^29^

Abnormal EEG findings were present in 59.4% (117/197) of patients with available data, and 45.3% (53/117) of these received a definitive diagnosis suggesting that epileptiform activity may serve as a phenotypic red flag for underlying genetic etiology. Of the 193 patients for whom imaging data were available, 99 (51.3%) had abnormal brain MRI. Structural abnormalities (e.g., white matter atrophy, matter changes, cortical dysplasia) were present in 43.8% (32/73) of patients with genetically diagnosed MRIs. This finding was not statistically significant in our analysis when compared to undiagnosed cases. Future studies with standardized neuroimaging protocols may better define this relationship.

**FIGURE 3:**
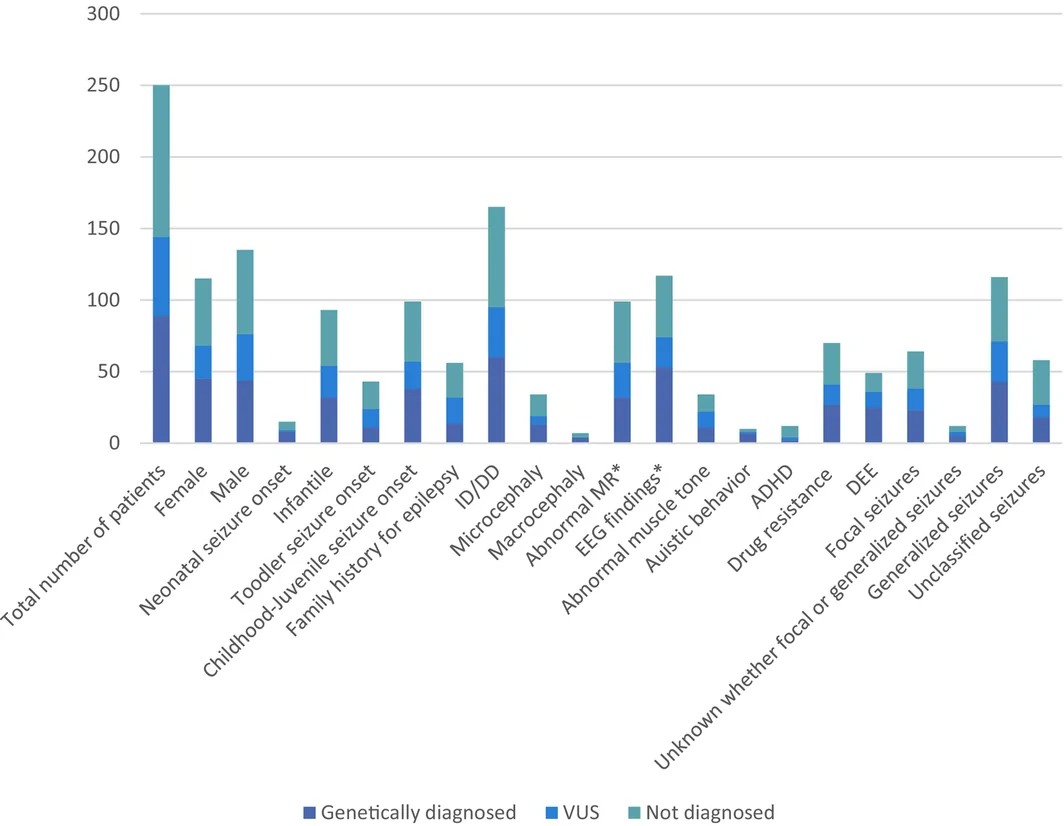
Phenotypic characteristics and diagnostic rates of the entire cohort. ID/DD, intellectual disability/ developmental delay; ADHD, attention‐deficit/hyperactivity disorder (DSM‐5 criteria).; DEE, developmental and epileptic encephalopathy; *MRI data available for 193/250 (77.2%) and EEG data available for 197/250 (78.8%) patients.

Molecular diagnosis rates based on the clinical presentation of our cohort are presented graphically in Figure 3.

### 4.4 The VUS conundrum and path to reclassification

A major challenge in clinical genomics remains the interpretation of variants of uncertain significance (VUS), identified in 22% of our cohort. While excluded from primary diagnostic yield calculations, many VUS occurred in genes with strong disease validity (*CNTNAP2*, *GAD1*, *RORB*, *GRIN2A*) and matched the patient's phenotype. For example, homozygous *GAD1* splice variants were identified in two siblings with infantile‐onset tonic seizures finding biologically plausible given *GAD1*'s role in GABA synthesis.

To address this, we propose a pragmatic, phenotype‐driven triage system to prioritize VUS for future re‐evaluation, specifically intended for research prioritization and not for immediate clinical diagnosis:

- Tier 1: VUS in genes with definitive ClinGen validity and strong phenotypic match → prioritize for targeted clinical surveillance, longitudinal follow‐up, and further evidence accrual (e.g., functional or segregation data) to clarify pathogenicity.
- Tier 2: Novel missense/splice variants in constrained genes (pLI ≥0.9) → prioritize for trio sequencing and segregation analysis. Genes with pLI ≥0.9 are highly intolerant to loss‐of‐function variants in population databases (e.g., gnomAD), indicating strong evolutionary constraint and increased likelihood that missense/splice variants in these genes are pathogenic. Trio sequencing is preferred over targeted parental segregation analysis for comprehensive variant detection—including de novo variants, compound heterozygosity, and uniparental disomy—but targeted segregation may be considered when resources are limited or a single high‐priority candidate variant exists.
- Tier 3: All other VUS entering longitudinal registries to enable future reclassification via data sharing (e.g., ClinVar, DECIPHER).

All VUS should undergo systematic re‐evaluation: Tier 1 variants at 6–12 months; Tier 2–3 variants at 2 years; and unresolved variants at ≥3 years or upon emergence of new functional evidence, additional affected family members, or significant phenotypic evolution.

Importantly, no VUS was used to alter clinical management in this cohort. Without trio analysis, de novo variant confirmation and segregation studies were limited, an acknowledged constraint that likely underestimates true diagnostic yield.

### 4.5 Precision medicine impact

As detailed in Section 3.5, genetic diagnosis directly informed therapeutic decisions in 12 of 89 diagnosed patients (13.5%): *SCN1A*‐positive cases (*n* = 9) avoided sodium channel blockers to reduce seizure exacerbation risk; *SLC2A1* variants (*n* = 2) prompted ketogenic diet trials with documented seizure reduction; and *MECP2* diagnosis (*n* = 1) enabled Rett syndrome‐specific surveillance. These outcomes mirror recent studies showing NGS‐driven management changes in 30–50% of pediatric epilepsy cases.^30^, ^31^ From a health economics perspective, early genomic diagnosis may reduce unnecessary diagnostic tests, hospitalizations, and ineffective therapies, though cost‐effectiveness analyses in Turkish settings remain needed.

### 4.6 Limitations and future directions

This study has key limitations:

1. No CNV or noncoding variant analysis: the absence of CNV analysis potentially limits diagnostic success, as combined detection of sequence and intragenic copy number variants has been shown to provide the sole molecular diagnosis in 5.1% of patients, significantly increasing the overall yield.^32^
2. The unavailability of biological parental samples for trio analysis necessitated a proband‐only interpretation, thereby limiting de novo variant confirmation and segregation studies.
3. EEG and MRI data were not uniformly available, reducing power for neurophysiological correlations.
4. Functional validation (e.g., electrophysiology for *SCN1A* variants) was not performed.

Future prospective studies integrating trio‐based genome sequencing, RNA‐seq for splice variant validation, and systematic deep phenotyping using Human Phenotype Ontology (HPO) terms are warranted to resolve VUS, uncover noncoding variants, and ultimately increase diagnostic yield beyond the 57.6% threshold observed here.

## AUTHOR CONTRIBUTIONS

Kadri Karaer: Conceptualization; methodology; supervision; data curation; writing—original draft; writing—review and editing. Derya Karaer: Data curation; formal analysis; investigation; writing—review and editing. Beste Kipçak Yüzbaşı: Investigation; resources; clinical phenotyping. İpek Şahin: Investigation; data curation; clinical phenotyping. Olcay Güngör: Investigation; clinical validation.

## FUNDING INFORMATION

This research did not receive any specific grant from funding agencies in the public, commercial, or not‐for‐profit sectors.

## CONFLICT OF INTEREST STATEMENT

The authors declare that they have no competing interests.

## PATIENT CONSENT

Written informed consent was obtained from the legal guardians of all participants for genetic testing and the use of anonymized clinical and genomic data for research purposes.

**Test yourself**

1. According to the findings in this pediatric epilepsy cohort, which of the following clinical features is a statistically significant predictor for establishing a definitive genetic diagnosis using next‐generation sequencing (NGS)?
  - Drug‐resistant epilepsy
  - Presence of intellectual disability/developmental delay (ID/DD)
  - Neonatal seizure onset
  - Abnormal structural findings on brain MRI
2. The study identified the PRRT2 c.649dup frameshift variant in four unrelated families and noted a high proportion (26%) of autosomal recessive diagnoses. Which underlying demographic factor in the Turkish population most strongly contributes to this distinct genetic architecture?
  - High prevalence of de novo mutations
  - High rates of consanguineous marriages
  - A predominant X‐linked inheritance pattern
  - A low diagnostic efficiency of Whole Exome Sequencing (WES)
3. Identifying a definitive molecular etiology can directly alter clinical management (clinical utility). In this study, what was the primary, actionable therapeutic change for patients diagnosed with SCN1A pathogenic variants?
  - Initiation of ketogenic diet therapy
  - Avoidance or discontinuation of sodium channel blockers
  - Evaluation for epilepsy surgery
  - Prescription of potassium channel openers

*Answers may be found in the* *Supporting Information*

## Supporting information


Appendix S1.

## Data Availability

The data that support the findings of this study are available from the corresponding author upon reasonable request. The raw genomic data are not publicly available due to privacy and ethical restrictions involving human participants.

## References

[1] Berkovic SF , Mulley JC , Scheffer IE , Petrou S . Human epilepsies: interaction of genetic and acquired factors. Trends Neurosci. 2006;29:391–397. 10.1016/j.tins.2006.05.009 16769131

[2] Al Anazi AH , Ammar AS , Al‐Hajj M , Cyrus C , Aljaafari D , Khoda I , et al. Whole‐exome sequencing of a Saudi epilepsy cohort reveals association signals in known and potentially novel loci. Hum Genomics. 2022;16:71. 10.1186/s40246-022-00444-6 36539902 PMC9764464

[3] Scheffer IE , Berkovic S , Capovilla G , Connolly MB , French J , Guilhoto L , et al. ILAE classification of the epilepsies: position paper of the ILAE Commission for Classification and Terminology. Epilepsia. 2017;58(4):512–521. 10.1111/epi.13709 28276062 PMC5386840

[4] Fisher RS , Cross JH , French JA , Higurashi N , Hirsch E , Jansen FE , et al. Operational classification of seizure types by the international league against epilepsy: position paper of the ILAE Commission for Classification and Terminology. Epilepsia. 2017;58(4):522–530. 10.1111/epi.13670 28276060

[5] Ottman R , Barker‐Cummings C , Leibson CL , Vasoli VM , Hauser WA , Buchhalter JR . Validation of a brief screening instrument for the ascertainment of epilepsy. Epilepsia. 2010;51:191–197. 10.1111/j.1528-1167.2009.02274.x 19694790 PMC2844922

[6] Mei D , Parrini E , Marini C , Guerrini R . The impact of next‐generation sequencing on the diagnosis and treatment of epilepsy in paediatric patients. Mol Diagn Ther. 2017;21:357–373. 10.1007/s40291-017-0257-0 28197949

[7] Helbig KL , Hagman Farwell KD , Shinde DN , Mroske C , Powis Z , Li S , et al. Diagnostic exome sequencing provides a molecular diagnosis for a significant proportion of patients with epilepsy. Genet Med. 2016;18(9):898–905. 10.1038/gim.2015.186 26795593

[8] Zou H , Zhang Q , Liao J , Zou D , Hu Z , Li B , et al. Diagnostic efficiency of exome‐based sequencing in pediatric patients with epilepsy. Front Genet. 2025;15:1496411. 10.3389/fgene.2024.1496411 39906551 PMC11790612

[9] Jiang A , Liu W , Liu Y , Zhang J . Efficacy of ketogenic diet therapy for pediatric drug‐resistant epilepsy with monogenic etiology: a single‐arm meta‐analysis. Nutr Rev. 2025;83(11):2104–2122. 10.1093/nutrit/nuaf140 40982721

[10] Natasha C , Lana L , Pegg E , Mohanraj R . Access to specialist epilepsy care by ethnicity and deprivation: analysis of a UK regional service journal of neurology, Neurosurgery & Psychiatry. J Neurol Neurosurg Psychiatry. 2024;95:A2. 10.1136/jnnp-2024-ABN.5

[11] Nashabat M , Al Qahtani XS , Almakdob S , Altwaijri W , Ba‐Armah DM , Hundallah K , et al. The landscape of early infantile epileptic encephalopathy in a consanguineous population. Seizure. 2019;69:154–172. 10.1016/j.seizure.2019.04.018 31054490

[12] Kablan A . Founder variants of the Turkish. Clin Genet. 2026;109(1):3–27. 10.1111/cge.70080 41074596

[13] Beniczky S , Trinka E , Wirrell E , Abdulla F , Al Baradie R , Alonso Vanegas M , et al. Updated classification of epileptic seizures: position paper of the international league against epilepsy. Epilepsia. 2025;66:1804–1823. 10.1111/epi.18338 40264351 PMC12169392

[14] Kwan P , Arzimanoglou A , Berg AT , Brodie MJ , Allen Hauser W , Mathern G , et al. Definition of drug resistant epilepsy: consensus proposal by the ad hoc task force of the ILAE commission on therapeutic strategies. Epilepsia. 2010;51(6):1069–1077. 10.1111/j.1528-1167.2009.02397.x 19889013

[15] Richards S , Aziz N , Bale S , Bick D , Das S , Gastier‐Foster J , et al. Standards and guidelines for the interpretation of sequence variants: a joint consensus recommendation of the American College of Medical Genetics and Genomics and the Association for Molecular Pathology. Genet Med. 2015;17(5):405–424. 10.1038/gim.2015.30 25741868 PMC4544753

[16] Lemke JR , Riesch E , Scheurenbrand T , Schubach M , Wilhelm C , Steiner I , et al. Targeted next generation sequencing as a diagnostic tool in epileptic disorders. Epilepsia. 2012;53:1387–1398. 10.1111/j.1528-1167.2012.03516.x 22612257

[17] Trump N , McTague A , Brittain H , Papandreou A , Meyer E , Ngoh A , et al. Improving diagnosis and broadening the phenotypes in early‐onset seizure and severe developmental delay disorders through gene panel analysis. J Med Genet. 2016;53(5):310–317. 10.1136/jmedgenet-2015-103263 26993267 PMC4862068

[18] Lindy AS , Stosser MB , Butler E , Downtain‐Pickersgill C , Shanmugham A , Retterer K , et al. Diagnostic outcomes for genetic testing of 70 genes in 8565 patients with epilepsy and neurodevelopmental disorders. Epilepsia. 2018;59(5):1062–1071. 10.1111/epi.14074 29655203

[19] Zou D , Wang L , Liao J , Xiao H , Duan J , Zhang T , et al. Genome sequencing of 320 Chinese children with epilepsy: a clinical and molecular study. Brain. 2021;144(12):3623–3634. 10.1093/brain/awab233 34145886 PMC8719847

[20] Castellotti B , Ragona F , Freri E , Messina G , Magri S , Previtali R , et al. Next‐generation sequencing in pediatric‐onset epilepsies: analysis with target panels and personalized therapeutic approach. Epilepsia Open. 2024;9:1922–1930. 10.1002/epi4.13039 39215763 PMC11450606

[21] Chen GH . Five cases of paroxysmal kinesigenic dyskinesia by genetic diagnosis. Exp Ther Med. 2015;9:909–912. 10.3892/etm.2014.2155 25667652 PMC4316949

[22] Kim SH , Seo J , Kwon SS , Teng L‐Y , Won DJ , Shin S , et al. Common genes and recurrent causative variants in 957 Asian patients with pediatric epilepsy. Epilepsia. 2024;65:766–778. 10.1111/epi.17857 38073125

[23] Kocaaga A , Yimenicioglu S . Identification of novel gene variants in children with drug‐resistant epilepsy: expanding the genetic Spectrum. Pediatr Neurol. 2023;139:7–12. 10.1016/j.pediatrneurol.2022.11.005 36493596

[24] Henry OJ , Ygberg S , Barbaro M , Lesko N , Karlsson L , Pérez LP , et al. Clinical whole genome sequencing in pediatric epilepsy: genetic and phenotypic spectrum of 733 individuals. Epilepsia. 2025;66:2966–2979. 10.1111/epi.18403 40183601 PMC12371643

[25] Koh HY , Smith L , Wiltrout KN , Podury A , Chourasia N , D'Gama AM , et al. Utility of exome sequencing for diagnosis in unexplained pediatric‐onset epilepsy. JAMA Netw Open. 2023;6(7):e2324380. 10.1001/jamanetworkopen.2023.24380 37471090 PMC10359957

[26] Kowkabi S , Yavarian M , Kaboodkhani R , Mohammadi M , Shervin Badv R . PCDH19‐clustering epilepsy, pathophysiology and clinical significance. Epilepsy Behav. 2024;154:109730. 10.1016/j.yebeh.2024.109730 38521028

[27] Tobiasz A , Hager M , Dębowska J , Jaunich A , Paprocka J . Tough to treat: what we know about managing PCDH19‐related epilepsy – systematic review. Seizure. 2025;132:98–109. 40934839 10.1016/j.seizure.2025.08.030

[28] Symonds JD , Zuberi SM , Stewart K , McLellan A , O'Regan M , MacLeod S , et al. Incidence and phenotypes of childhood‐onset genetic epilepsies: a prospective population‐based national cohort. Brain. 2019;142(8):2303–2318. 10.1093/brain/awz195 31302675 PMC6658850

[29] Stefanski A , Calle‐López Y , Leu C , Pérez‐Palma E , Pestana‐Knight E , Lal D . Clinical sequencing yield in epilepsy, autism spectrum disorder, and intellectual disability: a systematic review and meta‐analysis. Epilepsia. 2021;62(1):143–151. 10.1111/epi.16755 33200402 PMC7839709

[30] Haviland I , Daniels CI , Greene CA , Drew J , Love‐Nichols JA , Swanson LC , et al. Genetic diagnosis impacts medical Management for Pediatric Epilepsies. Pediatr Neurol. 2023;138:71–80. 10.1016/j.pediatrneurol.2022.10.006 36403551 PMC10099530

[31] McKnight D , Morales A , Hatchell KE , Bristow SL , Bonkowsky JL , Perry MS , et al. Genetic testing to inform epilepsy treatment management from an international study of clinical practice. JAMA Neurol. 2022;79(12):1267–1276. 10.1001/jamaneurol.2022.3651 36315135 PMC9623482

[32] Truty R , Patil N , Sankar R , Sullivan J , Millichap J , Carvill G , et al. Possible precision medicine implications from genetic testing using combined detection of sequence and intragenic copy number variants in a large cohort with childhood epilepsy. Epilepsia Open. 2019;4(3):397–408. 10.1002/epi4.12348 31440721 PMC6698688

