# Supplementary material for: Clinical utility and genetic landscape of exome sequencing in a large pediatric epilepsy cohort: Insights from a Turkish tertiary care center
Source: Epileptic Disord. 2026 Jul 3;28(4):1252–73. doi: 10.1002/epd2.70277 (PMC13499239; doi:10.1002/epd2.70277)
Supplement: Supplementary file 1 [file EPD2-28-1252-s001.docx]

**TEST YOURSELF**

**Answers**

**1. C**

Explanation: In this study, while ID/DD and drug resistance were highly prevalent, they did not significantly predict a genetic diagnosis. In contrast, neonatal seizure onset (p=0.03) and the presence of autistic features (p=0.04) were significantly associated with a higher diagnostic yield, emphasizing the importance of early NGS testing in these subgroups.

**2. B**

Explanation: Turkiye has a relatively high rate of consanguinity (approximately 20%), which increases the prevalence of autosomal recessive disorders and facilitates the emergence of population-specific recurrent variants or founder effects, such as the PRRT2 hotspot observed in this cohort.

**3. B**

Explanation: SCN1A variants were the most frequently identified etiology in this cohort. A crucial aspect of precision medicine in these patients (who frequently present with Dravet syndrome features) is the avoidance of sodium channel blockers (like carbamazepine) to prevent the exacerbation of seizures.
